# Iron load in the normal aging brain measured with QSM and $R_{2}^{*}$ at 7T: findings of the SENIOR cohort

**DOI:** 10.3389/fnimg.2024.1359630

**Published:** 2024-10-21

**Authors:** Miguel Guevara, Stéphane Roche, Vincent Brochard, Davy Cam, Jacques Badagbon, Yann Leprince, Michel Bottlaender, Yann Cointepas, Jean-François Mangin, Ludovic de Rochefort, Alexandre Vignaud

**Affiliations:** ^1^Université Paris-Saclay, CEA, CNRS, BAOBAB, Neurospin, Gif-sur-Yvette, France; ^2^CATI, US52-UAR2031, CEA, ICM, Sorbonne Université, CNRS, INSERM, APHP, Ile de France, France; ^3^VENTIO, Marseille, France; ^4^Université Paris-Saclay, CEA, Neurospin, UNIACT, Gif-sur-Yvette, France; ^5^Université Paris-Saclay, BioMaps, Service Hospitalier Frederic Joliot, INSERM, CEA, Orsay, France

**Keywords:** QSM, *R* ^*^_2_, healthy brain aging, brain iron, deep gray matter

## Abstract

**Background:**

Iron accumulates in the brain during aging and is the focus of intensive research as an abnormal load, particularly in Deep Gray Matter (DGM), is related to neurodegeneration. Magnetic Resonance Imaging (MRI) metrics such as Quantitative Susceptibility Mapping (QSM) and apparent transverse relaxation rate $R_{2}^{*}$ can be used to follow up iron *in vivo*. While the influence of age and sex on iron levels has already been reported, a careful consideration of neuronal risk factors, as well as for an enhanced sensitivity, is needed to define the normal evolution.

**Methods:**

QSM and $R_{2}^{*}$ at ultra-high field MRI are used to study iron in DGM using a carefully-characterized cohort of the healthy aging brain (SENIOR). Seventy-seven cognitively healthy elders (from 54 to 78 y/o) with clinical, biology, genetics, and cardiovascular risk factors careful evaluation. Differences linked with age, sex, cardiovascular risk factors and weight are studied.

**Results:**

Age and sex have an influence on the brain iron deposition measured by QSM and $R_{2}^{*}$ in a context of normal aging, without appearance of a pathological neurodegenerative process. Iron deposition shows higher values in the caudate and the putamen in older participants. Female participants present a higher level of iron in the amygdala, and males in the thalamus. Female participants also present differences in the accumbens, caudate and hippocampus when evaluating the joint age and sex effect. Participants with higher cardiovascular risk factors showed higher values of the iron, even without any impairment in their cognitive capability. An overweight is related with a higher iron load in the putamen for QSM and $R_{2}^{*}$ in female participants. We controlled that these modifications of iron deposition are not related to a specific profile in the genotype of ApoE loci.

**Conclusions:**

Establishing baseline values of QSM and $R_{2}^{*}$ as iron probes in the context of aging is essential to determine differences in the process of neurodegeneration. Age and sex of participants are important factors that affect brain iron normal values. On the other hand, the presence of cardiovascular risk factors, which can be associated with age related diseases, can also potentially be linked with the iron deposition in the brain.

## 1 Introduction

In developed countries, the proportion of the elderly population is increasing. Between 2021 and 2050, the ratio of Europeans aged above 65 should increase from 20.8% to 30%. In addition, the old age dependency ratio is projected to be 56.7% [European Community (EU), 2023]. This aging population is affected by multiple diseases and comorbidity such as hyperlipidemia, hypertension, diabetes, heart disease (Davis et al., 2011) and neurodegenerative diseases (Nichols et al., 2019).

The percentage of elderly people with an Alzheimer's disease (AD) increase with age. In the USA, it represents 5% of people aged between 65 and 74 y/o, and 13.1% of people aged between 75 and 84 y/o (Alzheimer's Association Report, 2023). In addition, Parkinson's disease (PD), the second most frequent neurodegenerative disease, represents 2% of people over 65 y/o. The major risk of neurodegenerative diseases, such as PD and AD, is aging, with only 5%–10% with an early onset before the age of 50. The development of new tools for the early diagnosis of neurodegenerative diseases remains a major challenge, as there is a lack of biomarkers for predicting brain disorders and mild cognitive impairment (Beach, 2017; Jeromin and Bowser, 2017), following up disease progression, supporting dementia affected people, or stratifying patients susceptible to respond to new therapies.

Iron is essential for the brain. In addition to its role in oxygen transport (hemoglobin), it is involved in several processes such as myelinization or neurotransmitters synthesis (Hare et al., 2013; Betts et al., 2016; Treit et al., 2021; Wang et al., 2017). Nevertheless, there is increasing evidence that iron accumulates heterogeneously in the brain throughout life and is involved in neurodegeneration. Indeed, an abnormal iron load in several brain regions, notably in deep gray matter (DGM) structures (such as putamen, caudate and globus pallidus), in older adults leads to cellular oxidative damages, inducing neuronal death (Ficiarà et al., 2022; Costello et al., 2004; Treit et al., 2021; Sousa et al., 2020). This iron-induced cell death, named ferroptosis, is the subject of intensive research (Dixon et al., 2012).

MRI is sensitive to iron and provides a means to quantify iron-related metrics, *in vivo* and in a non-invasive way (Ravanfar et al., 2021; Wang et al., 2022). Tissue (non heme) iron displays paramagnetic properties (Wood and Ghugre, 2008; Ropele and Langkammer, 2017) and, depending on its chemical form (*e.g*. ferrihydrite core of ferritin), can possess high magnetic susceptibility, which generates magnetic field perturbations (Schweser et al., 2016). MRI sequences can detect iron due to this physical property, allowing an estimation of its content in different tissues (Ravanfar et al., 2021). The effect of susceptibility in the tissue affects the apparent transverse relaxation time $T_{2}^{*}$ and can be easily estimated by means of 3D Multi-echo Gradient Echo (MGRE) (Keuken et al., 2017; Ropele and Langkammer, 2017). $T_{2}^{*}$ mapping analyzes the magnitude decay of the MRI signal. Interestingly, the phase of the signal also contains valuable information that reflects the tissue susceptibility effects by enabling to measure susceptibility-induced magnetic field deformations. This information is used by Quantitative Susceptibility Mapping (QSM) techniques to estimate a quantitative measure of the bulk susceptibility of a voxel (de Rochefort et al., 2009; Liu et al., 2012; Schweser et al., 2016; Ropele and Langkammer, 2017; Ruetten et al., 2019; Li Y. et al., 2021).

However, besides iron effects, the magnetic susceptibility-derived measures in the brain are also affected by some neuronal structures such as myelin, by calcification and by deoxyhemoglobin (Ficiarà et al., 2022). For instance, the presence of the myelin increase $R_{2}^{*}$ but decreases the relative susceptibility (Ficiarà et al., 2022). In DGM structures, which are mainly affected by neurodegeneration, the susceptibility effects from myelin and other paramagnetic materials are minor, therefore the measures are mostly determined by the iron load content (Ficiarà et al., 2022; Ravanfar et al., 2021).

MRI acquisition parameters have to be set properly, as they affect the sensitivity and specificity of subsequent iron deposition measurements, for both $R_{2}^{*}$ and for QSM (Wang et al., 2009). $R_{2}^{*}$ quantification depends notably on the magnetic field strength, since faster relaxation rates are obtained at higher magnetic field strengths (Peters et al., 2007; Deistung et al., 2013; Ropele and Langkammer, 2017). However, QSM measurements are not majorly affected by the strength of the magnetic field (Li Y. et al., 2021; Nikparast et al., 2022). The magnetic property measured by QSM is inherent to the tissue and therefore independent of the field strength (Li et al., 2018; Li Y. et al., 2021).

Ultra-high magnetic field (UHF) strengths MRI (>3T) provide a higher signal-to-noise ratio (SNR). This allows the acquisition of images at a higher resolution within clinical-feasible scan times without the mitigation of the sensitivity (Vachha and Huang, 2021). However, these acquisitions can also be more vulnerable to artifacts or effects from strong field variations due to the air/tissue interface that need to be accounted for (Vachha and Huang, 2021; Wang et al., 2022; Daval-Frérot et al., 2022). Despite the challenge this presents, UHF has provided improved $T_{2}^{*}$ contrast, which can be helpful in pathological situations (*e.g*. in differentiating histological types of cortical multiple sclerosis lesions) (Cohen-Adad et al., 2011).

Image resolution also plays a role in $R_{2}^{*}$ and QSM values. High image resolution decreases partial volume effects, leading to a higher specificity (especially in the presence of small veins within a voxel) (Haacke et al., 2015). This is particularly important for compact structures such as small deep nuclei. In fact, slice thickness has been reported to reduce by 10% the mean susceptibility for small structures (Li Y. et al., 2021)

Several studies have analyzed iron load *in vivo* using $R_{2}^{*}$ and/or QSM, either from the normal aging perspective or with a focus on neurodegenerative diseases. Higher QSM values for the regions implicated in diseases, especially DGM, have been reported (see Ravanfar et al., 2021 for an extensive review).

Iron accumulation with age in healthy subjects has also been reported previously, with the goal to describe normal reference values. This is of utmost importance for future applications in the evaluation of deviations in diseases by demonstrating higher values in several subcortical nuclei (Siemonsen et al., 2008; Li et al., 2014; Acosta-Cabronero et al., 2016; Treit et al., 2021). These studies differ either in terms of cohort characteristics (age range and size) or MRI acquisition parameters (magnetic field strength and image resolution, often larger than 1 mm isotropic). Moreover, a comprehensive description of the cohort's health condition is often absent (for instance, no information on risk factors of the subjects, despite their healthy cognitive state, is given). Also, these studies have been mainly performed at 3T, at rather low spatial resolution (for review, see Madden and Merenstein, 2023).

Slight higher values of iron load in DGM during old age have been described for QSM and $R_{2}^{*}$ in lifespan studies (Li et al., 2014; Treit et al., 2021). This trend has also been described for young and middle-aged adults using QSM (Burgetova et al., 2021), as well as for elders (Gong et al., 2015; Li Y. et al., 2021; Li et al., 2023; Liu et al., 2016; Persson et al., 2015; Poynton et al., 2014). By means of $R_{2}^{*}$ alone, correlations with age and higher values in certain regions of middle-aged adults and elder subjects have also been reported (Holz et al., 2022; Daugherty and Raz, 2016; Pirpamer et al., 2016).

QSM and Field-Dependent Relaxation Rate Increase (FDRI) have also shown higher iron concentrations in certain regions in older participants (Bilgic et al., 2012). In the spirit of documenting the differences in iron load measured by QSM, age-specific atlases have been proposed, reporting also higher values in specific regions (Lao et al., 2023; Zhang et al., 2018). Iron load differences through aging have also been described longitudinally for middle-aged and elder adults, evidencing a correlation between age and DGM when comparing two time-points (Li J. et al., 2021). Moreover, the consistency of QSM measurements over different vendor machines has been reported, whose results are also in agreement with the literature, showing a positive correlation with age (Li Y. et al., 2021).

Furthermore, although less often, 7T data has also been used to address this matter. At a higher image resolution [voxel size ≤ (0.8*mm*)^3^], the agreement between $R_{2}^{*}$ and QSM, which describes age-related differences due to iron accumulation in subcortical regions, has also been reported (Betts et al., 2016; Keuken et al., 2017).

To date, there is no study using UHF that focuses exclusively on healthy cognitive aging from middle to very old age using a large, well-characterized cohort and accounting for common risk factors that are frequent in the aging population (such as hypertension, hyperlipidemia or diabetes).

In this work, the focus relies on quantifying QSM and $R_{2}^{*}$ on healthy brain aging at old ages using UHF-MRI, leveraging on the well-characterized SENIOR database. The latter documents biological, psychological, and imaging data (including amyloid PET imaging) from healthy older adults. This allows for an accurate description of the population, confirming the absence of neurodegenerative diseases and limiting the sources of variation due to inherent risk factors.

Using the SENIOR database high-resolution imaging data acquired at 7T, $R_{2}^{*}$ and QSM values are computed for the DGM. From these regions, we identified those that present differences: (i) regarding age, providing also their normal values; (ii) with respect to sex; (iii) related with an increased cardiovascular risk factor; and (iv) linked with overweight. The effect of the presence of ϵ4 allele of apolipoprotein E (ApoE) was also evaluated, as presumed genetic risk factor in the malfunction of brain iron homeostasis (Wood, 2015).

## 2 Materials and methods

### 2.1 Participants

It includes UHF 7T magnetic resonance imaging acquisitions for high-resolution brain characterization. Seventy-seven volunteers data were available at the time of this work, from acquisitions after a protocol update (reason why only one point per participant is included) and they were selected for the analysis. The details for 77 participants (54–78 years old, 37 males/40 females) are presented in the Supplementary Tables S1–S6.

### 2.2 Image acquisition

The imaging data were acquired using a 7T MRI system (Magnetom 7 Tesla investigational device, Siemens Healthineers, Erlangen, Germany) equipped with a whole body gradient (maximum gradient strength Gmax 100 mT/m, slew rate T/m/s) 1Tx/32Rx Nova Medical head coil. A high-resolution multi-gradient-echo acquisition (MGRE) was performed [acquisition time TA = 9:48 min, field of view FoV = 256 mm, voxel size = 0.8 mm^3^ isotropic, repetition time TR = 37 ms, echo time TE = 1.68 ms, ΔTE = 3.05 ms, number of echoes = 10, flip angle = 30°, acceleration factor Generalized Autocalibrating Partially Parallel Acquisitions (GRAPPA) = 3, 196 sagittal partitions, bandwidth = 740 Hz/px, monopolar readouts] as well as $B_{1}^{+}$ and *B*_0_ maps for calibration and correction purposes. A *T*_1_-weighted MP2RAGE was also acquired (TR = 6,000 ms; TE = 2.96 ms; voxel size = 0.75 mm^3^ isotropic). MGRE phase data were reconstructed using Virtual Coil Combination (VCC) (Santin, 2018).

### 2.3 QSM and $R_{2}^{*}$ reconstruction and analysis

We implemented a pipeline to compute $R_{2}^{*}$ and QSM maps and extract the values from regions of interest (ROIs). It uses 3D MGRE DICOM data as input and outputs the computed maps, as well as the ROIs segmented values. Notably, it incorporates a phase filtering step that reduces the effect of phase artifacts in the input data. Additionally, it was implemented in a secured cloud environment. A detailed description of the pipeline and its implementation is provided in the Supplementary Section 3.

### 2.4 Statistical analysis

The following DGM structures were studied: accumbens, amygdala, caudate, globus pallidus, hippocampus, putamen and thalamus. The analysis steps described below are applied to QSM and $R_{2}^{*}$ data independently. For each subject and ROI only the high iron content part of the region was considered, *i.e*. values contained within [μ−2σ, μ+2σ], as described in previous work (Liu et al., 2016). Then, the average values are computed for further analyses, as a means to represent each ROI value (Treit et al., 2021; Siemonsen et al., 2008; Cheng et al., 2020).

First, in order to obtain a summary of the relationship between the different DGM values (from $R_{2}^{*}$ and QSM) and the age for female and male participants, we performed a linear regression using least-squares between these two variables, using Scipy package version 1.9.3 and Python version 3.10.9. The regressions were performed using the age as a continuous independent variable and either the QSM or $R_{2}^{*}$ values as dependent variable. Then, we looked into a possible relationship between the volume of the ROIs and the iron level measured by means of QSM and $R_{2}^{*}$. In order to do that, for each region, we computed the correlation between its volume obtained from the volBrain^1^ pipeline (Manjón and Coupé, 2016) (based on the *T*_1_-w MP2RAGE) and the iron level measures given by the two proxies. The volume measure was also normalized by the Total Intracranial Volume (TIV).

Finally, we looked if among the studied regions, there is any for which the iron level (measured by means QSM and $R_{2}^{*}$) is significantly different between groups, given a specific population parameter. The differences between these groups were evaluated by means of a Mann–Whitney *U*-test (McKnight and Najab, 2010). The groups were defined for six specific parameters, described in the paragraphs below and the differences were evaluated for the QSM and $R_{2}^{*}$ values for each ROI. As we perform multiple comparisons for seven ROIs and six parameters giving a total of 16 pairwise comparisons, in order to take account of type I risk, we computed the Bonferoni correction for the *p*-values, by a factor of 112. Both corrected and uncorrected *p*-values are presented for a more comprehensive view of the results.

#### 2.4.1 Demographic-based groups

To investigate ROI's values differences regarding demographics, the population was studied in terms of age and sex. First, the population was subdivided into three homogeneous groups based on their age, according to the ranges: [54, 62], [62, 69], and [69, 78] y/o with 24, 26 and 27 individuals, respectively. In order to evaluate the influence of the participant's sex on QSM and $R_{2}^{*}$ quantification, the whole population was grouped into male and female participants. The joint effect of age and sex was also tested by using both subdivisions described above.

#### 2.4.2 ApoE ϵ4-based groups

The presence of ApoE ϵ4 genotype is a major concern in the aging population, with an increasing risk of developing a neurodegenerative disease. Therefore, the effect of the presence of the ApoE ϵ4 allele was tested as well. Heterozygote participants for the ϵ4 allele (16 subjects) were compared with the rest of the population that does not present it.

#### 2.4.3 Cardiovascular risk score-based groups

Premature cardiovascular disease has been described to be associated with an early cognitive decline (Jiang et al., 2023). Moreover, smoking, which has a crucial role in the development of cardiovascular disease, has also been described to be related with higher iron load in the DGM (Pirpamer et al., 2016). We evaluated the impact on QSM and $R_{2}^{*}$ values of factors that increase the risk of developing a cardiovascular disease, and therefore also entail neurological effects, by using a cardiovascular risk score (CRS, from 0: low risk to 5: high risk) that summarizes these factors (Haeger et al., 2020) (see Supplementary Section 4).

#### 2.4.4 Body mass index-based groups

Differences in QSM and $R_{2}^{*}$ related to body weight were evaluated by means of the Body Mass Index (BMI). The BMI has also been described to be related with an iron overload in the brain, measured using $R_{2}^{*}$ (Pirpamer et al., 2016; Holz et al., 2022). We therefore evaluated the impact of overweight (BMI > 25) on the values obtained from QSM and $R_{2}^{*}$ in male and female participants separately.

## 3 Results

### 3.1 Main population characteristics

The population involved in our study were aged 54–78, divided in three age groups 54–62, 62–69, and 69–78, with 24, 26, and 27 participants respectively. The whole population was equally divided between males and females, with 37 and 40 participants, respectively. In the group 69–78, the number of female participants (17) was higher than in the others groups, probably due to demographics. We observed a progressive increase of hypertension frequency to reach 50 and 29.4% in males and females over 69 years old, respectively. We noted no significant increase with age of the ratio between high-density lipoprotein (HDL), low-density lipoprotein (LDL) and triglycerides. Notably, 11.1%–22.2% of female participants in the different age groups have been considered to present depression (geriatric depression scale GDS ≥10) whereas in male groups, nearly no participant experiences this state. We noticed that no severely depressed participants were present in our cohort (GDS >20, see Supplementary Table S1). As anemia is a common comorbidity of aging populations and is related to iron load in cells, we controlled the stability of hemoglobin, mean corpuscular volume (MCV), mean corpuscular hemoglobin (MCH) and mean corpuscular hemoglobin concentration (MCHC). As expected, hemoglobin and hematocrit levels of female participants are lower than those of male, with all values being in the normal range for each sex. Importantly, we controlled that our population is cognitively unimpaired (see Supplementary Table S1). All participants presented a normal mini mental state MMS examination and a normal Mattis rating scale.

### 3.2 QSM and $R_{2}^{*}$ maps

**Figure 1:**
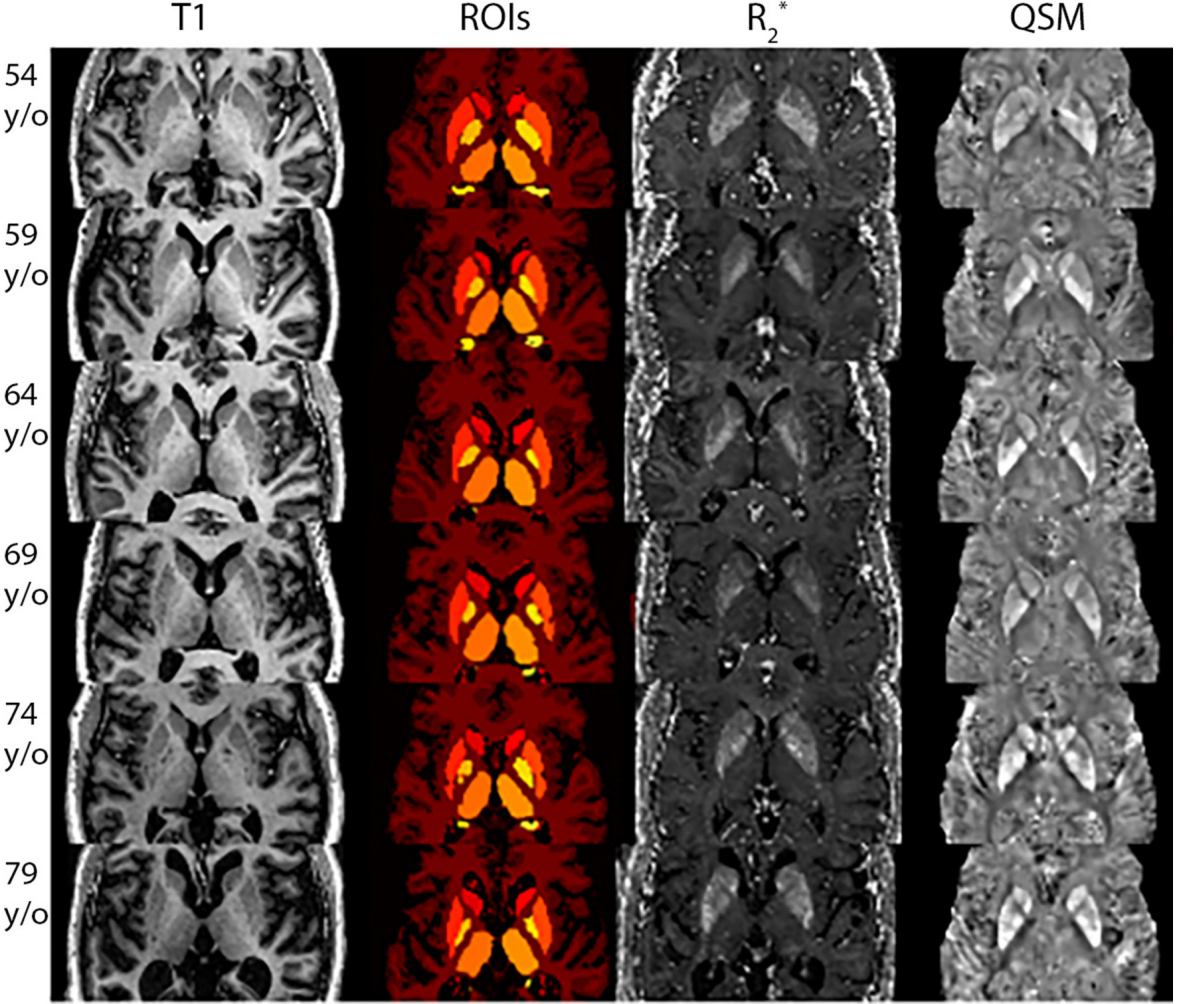
Uniform Denoised (UNIDEN) *T*_1_-w, ROIs, $R_{2}^{*}$ and QSM for selected participants with different ages. Each row presents the results for each participant, going from the youngest to the oldest. The first column presents the Uniden *T*_1_-w image used to segment the DGM and obtain the ROIs, displayed in the second column. The third and fourth column show the results for $R_{2}^{*}$ and QSM reconstructions, respectively.

The application of the developed pipeline (described in Supplementary Section 3) generated reliable QSM and $R_{2}^{*}$ maps. An example of these results are displayed in Figure 1, for six participants of different ages. It can be seen that some regions known to present a higher iron load (*e.g*. the putamen) show higher intensities for QSM and $R_{2}^{*}$.

### 3.3 Statistical analysis results

From the QSM and $R_{2}^{*}$ maps, inter-hemispheric mean differences were first evaluated, for which no significant right/left difference was obtained. Therefore, the average values for the contralateral ROIs together were computed and considered in the following.

In order to quantify the relationship between the age and the iron load, we computed the linear regression for each DGM ROIs for female and male participants (see Table 1 for the linear regression coefficients and Supplementary Figure S3 for the linear regression plots). This helps to model the trend of the iron load quantification values, as well as predicting their normality in new participants. We found for the caudate a positive slope of iron quantification by QSM of 0.63 (±0.26) ppb per year for female participants (*p* = 0.02) and 1.09 (±0.47) ppb per year for male participants (*p* = 0.03). Following the same trend, the slope for $R_{2}^{*}$ quantification was 0.36 (±0.13) *s*^−1^ per year (*p* = 0.01) and 0.43 (±0.19) *s*^−1^ per year (*p* = 0.03) for female and male participants, respectively. For the putamen, a greater slope was measured for QSM 1.25 (±0.33) ppb per year (*p* < 0.01) and 1.5 (±0.54) (*p* < 0.01) ppb per year for female and male participants, respectively. As for $R_{2}^{*}$, we obtained the values of 0.69 (± 0.21) *s*^−1^ per year (*p* < 0.01) and 0.72 (±0.29) *s*^−1^ per year (*p* = 0.02) for female and male participants, respectively. For the hippocampus, we evidenced a negative slope for the iron load measured by $R_{2}^{*}$ of 0.08 (±0.04) *s*^−1^ per year (*p* = 0.04) for female participants. We also noticed a negative slope of the iron load measured by QSM of 1.4 (±0.57) ppb per year (*p* = 0.02) in the accumbens of female participants. Moreover, we also noted in $R_{2}^{*}$ values of female participants a negative slope for the amygdala [0.09 ± 0.04 *s*^−1^ per year (*p* = 0.05)] and a positive slope for the globus pallidus (0.86 ± 0.4 *s*^−1^ per year (*p* = 0.04)). As for the male participants, we noticed that $R_{2}^{*}$ values for the accumbens [0.26 ± 0.1 *s*^−1^ per year (*p* = 0.02)], and the amygdala [0.12 ± 0.05 *s*^−1^ per year (*p* = 0.05)] show a positive slope.

**Table 1:** Linear regression results for QSM and $R_{2}^{*}$.

		**QSM**			$R_{2}^{*}$		

**ROI**	**Sex**	**Slope** ±**SE (ppb/year)**	**Intercept** ±**SE (ppb)**	*p* **-value**	**Slope** ±**SE (***s*^−1^**/year)**	**Intercept** ±**SE (***s*^−1^**)**	*p* **-value**
Caudate	♀	0.63 ± 0.26	–4.97 ± 17.66	0.02	0.36 ± 0.13	21.4 ± 8.89	0.01
	♂	1.09 ± 0.47	–31.56 ± 30.48	0.03	0.43 ± 0.19	20.33 ± 12.26	0.03
Putamen	♀	1.25 ± 0.33	–49.68 ± 22.49	≤ 0.01	0.69 ± 0.21	11.67 ± 14.49	≤ 0.01
	♂	1.5 ± 0.54	–63.11 ± 34.85	≤ 0.01	0.72 ± 0.29	13.42 ± 18.79	0.02
Thalamus	♀	–0.09 ± 0.19	–10.64 ± 12.58	0.62	–0.07 ± 0.07	42.67 ± 4.67	0.31
	♂	–0.02 ± 0.24	–8.23 ± 15.21	0.94	–0.02 ± 0.07	39.83 ± 4.3	0.9
Globus pallidus	♀	0.06 ± 0.56	85.42 ± 37.95	0.92	0.86 ± 0.4	36.23 ± 27.06	0.04
	♂	0.54 ± 0.59	50.69 ± 38.37	0.37	0.0 ± 0.45	88.5 ± 29.12	0.99
Hippocampus	♀	–0.31 ± 0.18	5.2 ± 12.41	0.1	–0.08 ± 0.04	36.17 ± 2.51	0.04
	♂	–0.14 ± 0.2	–5.02 ± 12.68	0.48	0.03 ± 0.06	29.4 ± 3.6	0.58
Amygdala	♀	–0.51 ± 0.34	17.88 ± 23.09	0.14	–0.09 ± 0.04	32.56 ± 2.94	0.05
	♂	–0.18 ± 0.29	–12.78 ± 18.51	0.54	0.12 ± 0.05	19.23 ± 3.39	0.03
Accumbens	♀	–1.4 ± 0.57	87.74 ± 38.87	0.02	0.12 ± 0.08	24.46 ± 5.23	0.14
	♂	–0.14 ± 0.54	–3.17 ± 35.02	0.8	0.26 ± 0.1	17.15 ± 6.69	0.02

Results from the linear regression analysis for the QSM and $R_{2}^{*}$ maps with respect to age. For each region, the results are informed for the female and male participants. These results include: the slope and intercept and their respective standard error (SE).

In order to evaluate a possible relationship between the region's volume and the level of iron, we performed a correlation between these two measures. No correlation was found for any of the regions, either for the non-normalized volumes and the normalized volumes by the TIV.

#### 3.3.1 Age-based differences

**Figure 2:**
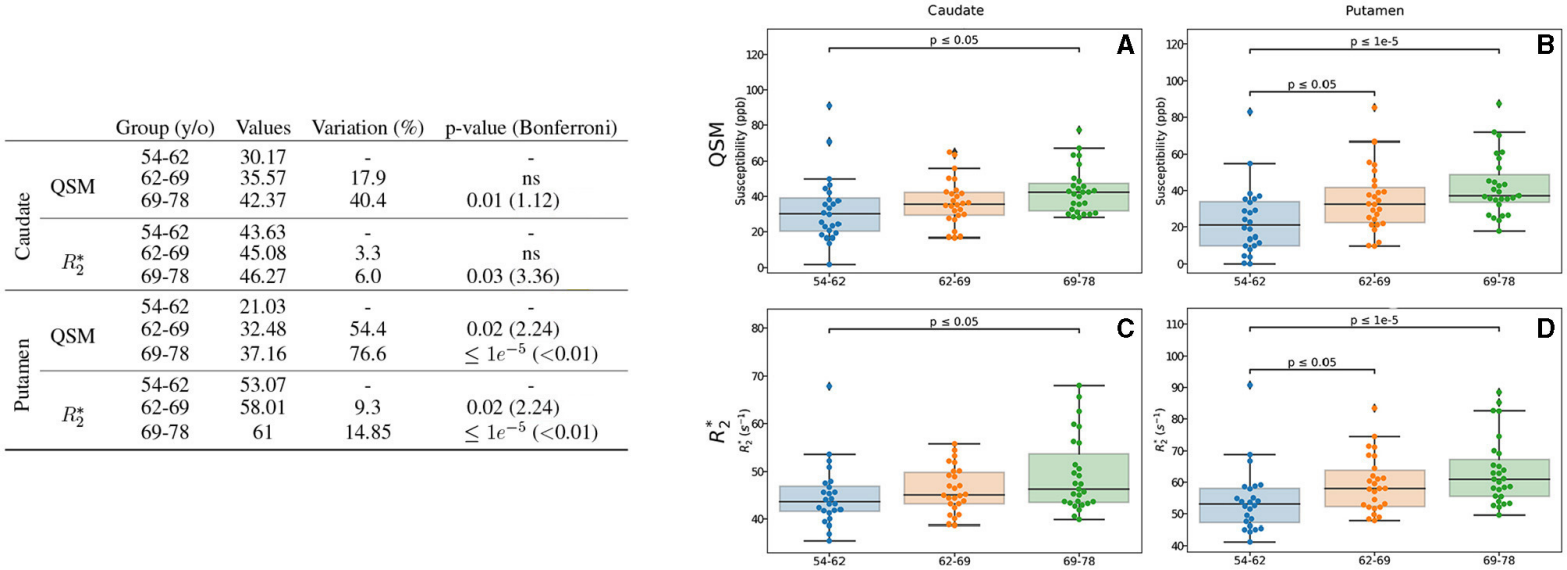
Results for the analysis of the age-based groups. Only the regions exhibiting differences for QSM and/or $R_{2}^{*}$ are presented. The table on the left summarizes for each region, metric and age-range: the median value, the variation percentage existing with respect to the younger group, and the *p*-value from the Mann–Whitney *U*-test. Bonferroni corrections for the *p*-value are shown in parentheses. On the right are displayed the box plots with the *p*-values before correction (for the different groups) for QSM **(A, B)** in *ppb* and $R_{2}^{*}$(C, D) in *s*^−1^ for caudate **(A, C)**, putamen **(B, D)**.

The results from analysis of QSM and $R_{2}^{*}$ with respect to age are displayed in Figure 2, only for the ROIs showing differences. We observed differences of intracerebral iron measured by QSM in two regions: caudate and putamen. In the caudate, higher values of an 18 and 40% for 62–69 and 69–78 y/o groups, respectively, were observed. The difference between the first and the last group presents a *p*-value = 0.01. For $R_{2}^{*}$, a similar but limited profile is observed (6%, *p* = 0.03). In the putamen, we observed higher values of a 76.6% (*p* < 0.01 after Bonferroni correction) for QSM. As before, the difference in $R_{2}^{*}$ values remain modest (being 14.85% higher), but significant with a *p* < 0.01 after Bonferroni correction.

#### 3.3.2 Sex-based differences

**Figure 3:**
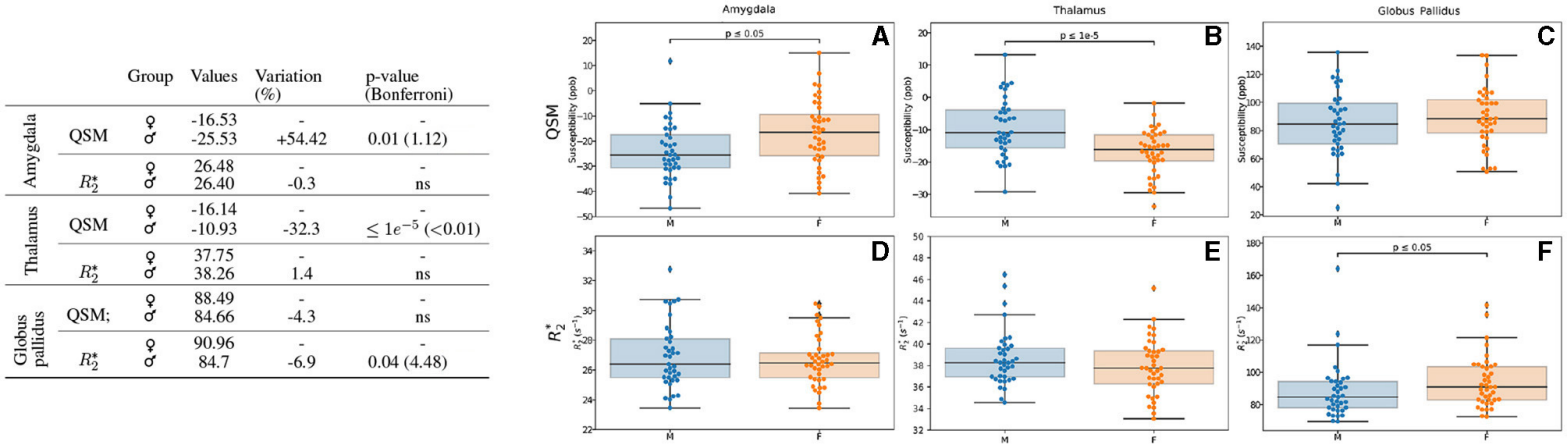
Results for the analysis of the sex-based groups. Only the regions exhibiting differences for QSM and/or $R_{2}^{*}$ are displayed. The table on the left summarizes for each region, metric and sex: the median value, the variation percentage existing for males as compared to females, and the *p*-value from the Mann–Whitney *U*-test. The Bonferroni correction for the *p*-values is shown in parentheses. On the right can be seen the box plots without the Bonferroni correction for the amygdala for QSM **(A)** and $R_{2}^{*}$(D), for the thalamus for QSM **(B)** and $R_{2}^{*}$(E) and the globus pallidus for QSM **(C)** and $R_{2}^{*}$(F). QSM values are expressed in *ppb* and $R_{2}^{*}$ values are expressed in *s*^−1^.

We observed that the participant's sex has a notable influence in iron load quantified by QSM in the amygdala and thalamus regions (Figure 3). Higher values in the amygdala of female participants (54%, *p* = 0.01) and in the thalamus of male participants (32%, *p* < 0.01 after Bonferroni correction) were detected. We noted a modest higher value of $R_{2}^{*}$ signal in the globus pallidus region of the brain in the male participants (7%, *p* = 0.04).

#### 3.3.3 Age- and sex-based differences

**Figure 4:**
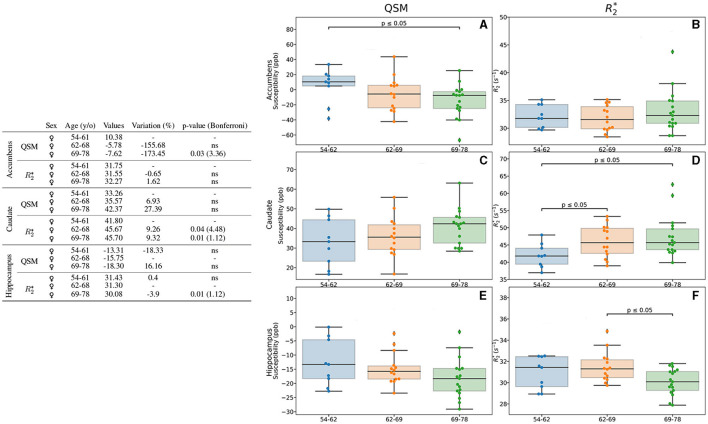
Results of the analysis for the age- and sex-based groups. Only the regions exhibiting differences for QSM and/or $R_{2}^{*}$ for the female group are displayed. The table on the left summarizes for each region, metric, age group and sex: the median value, the variation percentage existing from one group to the other, and the *p*-value from the Mann–Whitney *U*-test. The Bonferroni correction for the *p*-values is shown in parentheses. On the right can be seen the box plots without the Bonferroni correction for the accumbens for QSM **(A)** and $R_{2}^{*}$(B), for the caudate for QSM **(C)** and $R_{2}^{*}$(D), the hippocampus for QSM **(E)** and $R_{2}^{*}$(F). QSM values are expressed in *ppb* and $R_{2}^{*}$ values are expressed in *s*^−1^.

**Figure 5:**
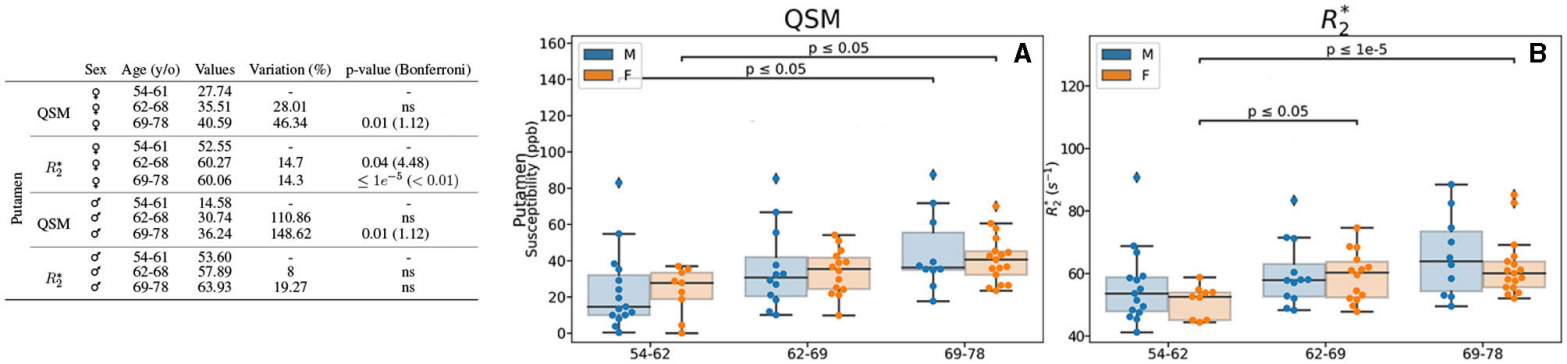
Results for the analysis of the age- and sex-based groups. Only the region (putamen) exhibiting differences for QSM and/or $R_{2}^{*}$ for both female and male groups is displayed. The table on the left summarizes for the region, metric, age group and sex: the median value, the variation percentage existing from one group to the other, and the *p*-value from the Mann–Whitney *U*-test. The Bonferroni correction for the *p*-value is shown in parentheses. On the right can be seen the box plots without the Bonferroni correction for the region QSM **(A)** and $R_{2}^{*}$(B). QSM values are expressed in *ppb* and $R_{2}^{*}$ values are expressed in *s*^−1^.

In addition to the separate analysis by age (Section 3.3.1) and sex (Section 3.3.2), we analyzed the combined effect of these two parameters in the measures of the iron load quantification by means of QSM and $R_{2}^{*}$ (Figures 4, 5). We found differences in the accumbens, caudate and hippocampus of female participants. In the accumbens, lower values regarding age (173%, *p* = 0.03) were observed with QSM quantification (Figure 4A), whereas $R_{2}^{*}$ measurement remains stable (Figure 4B). In the caudate, both QSM and $R_{2}^{*}$ show higher values for older participants (Figures 4C, D). It can be observed higher values in the 69–78 y/o group regarding the 54–62 y/o one (27.39% higher), while $R_{2}^{*}$ ones are higher by a 9.32% (Figure 4D). Although QSM higher values remain a trend, $R_{2}^{*}$ values are higher when analyzing the 54–62 y/o group vs. both 62–69 (*p* = 0.04) and 69-78 (*p* = 0.01) y/o groups (Figure 4D). We noticed in the hippocampus, by means of $R_{2}^{*}$, a modest decreasing difference of 3.9% between the 62–69 y/o and 69–78 y/o groups (Figure 4F).

Regarding the putamen, in addition to the previously mentioned higher values of QSM and $R_{2}^{*}$ regarding age when considering female and male participants together (Figure 2), we observed also higher values with age in both male and female groups when analyzed independently (Figures 5A, B). Interestingly, we observed that in the putamen of female participants QSM quantification reaches higher values of a 46.34% (*p* = 0.01) whereas for male participants it reaches 148.62% (*p* = 0.01). As for $R_{2}^{*}$ the difference is lower (14.3%), but it remains significative after Bonferroni correction (*p* < 0.01).

#### 3.3.4 ApoE ϵ4 group differences

The distribution of the ApoE allele in the population is detailed in Supplementary Table S6. Sixteen participants (20.78%) present at least one allele ϵ4, with a female/male ratio of 1.33 (one participant was ϵ4 homozygote). With respect to the presence of only one ApoE ϵ4 allele, it was found that it does not modify significantly QSM and $R_{2}^{*}$ in all tested regions.

#### 3.3.5 Cardiovascular risk score group differences

Regarding the CRS, participants presented only scores 0 (40 subjects), 1 (20 subjects) and 2 (17 subjects), out of a maximum of 5, which represents a higher risk of developing cardiovascular diseases. This distribution is coherent with our inclusion criteria and the aim of this study.

**Figure 6:**
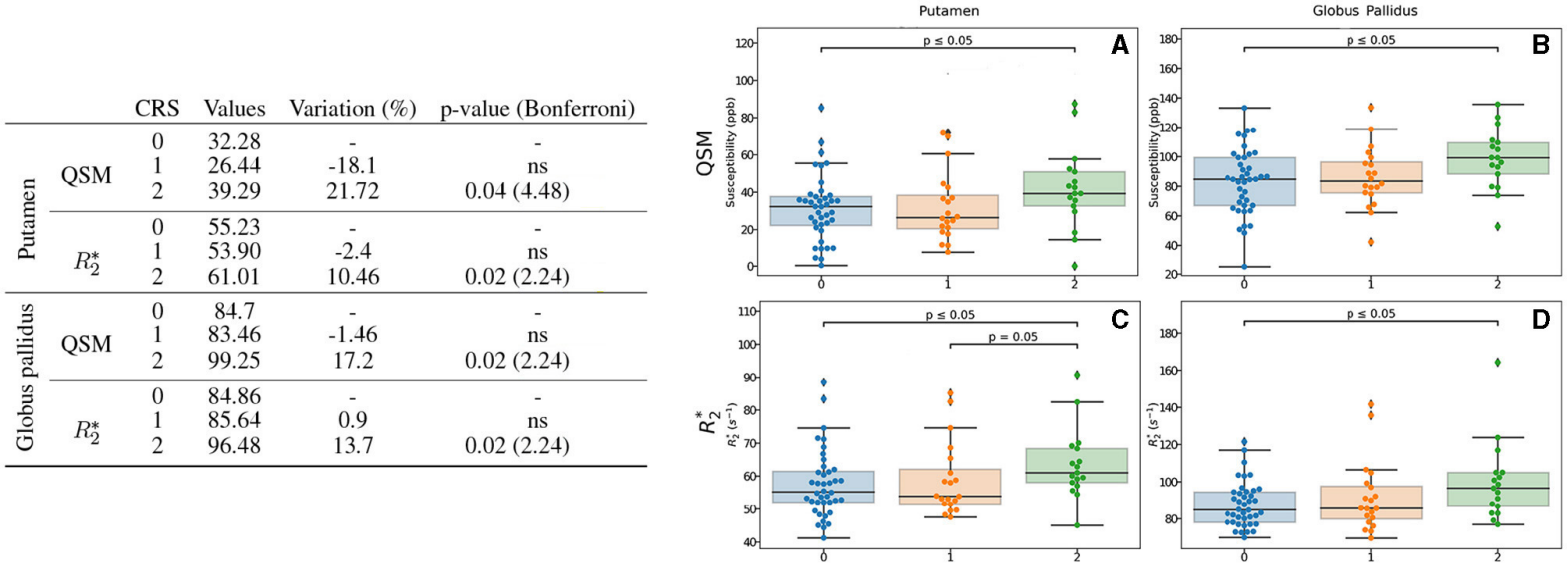
Results for the CRS-based group analysis. Only the regions exhibiting differences regarding CRS for QSM and/or $R_{2}^{*}$ are displayed. The table on the left summarizes for each region, metric and CRS: the median value, the variation percentage existing with respect to the lower score, and the *p*-value from the Mann–Whitney *U*-test. Bonferroni correction for the *p*-value is shown in parentheses. On the right can be seen the box plots without the Bonferroni correction for the putamen for QSM **(A)** and $R_{2}^{*}$(C), for the globus pallidus for QSM **(B)** and $R_{2}^{*}$(D). QSM values are expressed in *ppb* and $R_{2}^{*}$ values are expressed in *s*^−1^.

Interestingly, we observed some difference between participants without (CRS = 0) or with very low score (CRS = 1) of cardiovascular risk and participants that scored 2 as factor of cardiovascular risk (CRS = 2). Iron measures in the putamen and globus pallidus quantified by QSM show higher values in a 21.7% and 17.2%, with a *p*-value of 0.04 and 0.02 respectively (Figure 6). It was evidenced that for $R_{2}^{*}$, it follows the same profile as QSM but with a lower percentage of 10.46% for putamen and 13.7% for globus pallidus, respectively, with a *p*-value of 0.02 for both.

#### 3.3.6 BMI-based groups

**Figure 7:**
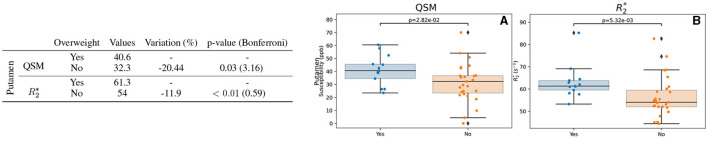
Results for the BMI-based group analysis. Only the region (putamen) exhibiting differences regarding BMI for QSM and/or $R_{2}^{*}$ is displayed. The table on the left summarizes metric and BMI category (considered overweight: Yes or not: No): the median value, the variation percentage existing with respect to an overweight, and the *p*-value from the Mann–Whitney *U*-test. Bonferroni correction for the *p*-value is shown in parentheses. On the right can be seen the box plots without the Bonferroni correction for QSM **(A)** and $R_{2}^{*}$(B). QSM values are expressed in *ppb* and $R_{2}^{*}$ values are expressed in *s*^−1^.

Regarding the BMI, 29 participants were considered to be overweight (BMI >25). We observed some differences between overweight and non-overweight in the putamen for female participants for both QSM and $R_{2}^{*}$ (Figure 7). The modification of QSM and $R_{2}^{*}$ values does not pass Bonferroni correction.

A summary of the significant differences regarding each metric is presented in the Supplementary Table S10.

## 4 Discussion

In this paper, we presented the study of the brain iron concentration differences in the normal aging by means of QSM and $R_{2}^{*}$, which serve as proxies for such differences, and using a well-characterized cohort with high quality data and a state-of-the-art reconstruction pipeline. The SENIOR cohort presents a rich database of particularly healthy and cognitive unimpaired elderly, documented by a complete neuropsychological evaluation and absence of amyloid deposits (on PET imaging). Medical history, blood pressure, clinical, biological and genetic susceptibility evaluations are also available, ensuring low or asymptomatic presence of main comorbidities, which are common to old age.

Due to the robustness of the pipeline, we were able to exploit the high quality MRI acquisitions at 7T, which provided a means to improve the description of the iron load differences in healthy cognitive aging. This along with the advanced and centralized image processing pipeline, which allowed us to improve the quality of the QSM and $R_{2}^{*}$ maps, enabled us to establish a trend of normality for our cohort, according to age and sex for specific regions. For both methods, our results are consistent with the literature, specially for the regions that are known to particularly accumulate iron (*i.e*. putamen and caudate). In fact, the putamen is an iron-rich region whose iron deposition increase starts in the middle age, which has been evidenced also by means of susceptibility measurements (Khan et al., 2012). Linear regression models and correlations have been used to describe the relationship between QSM values and age for these regions for participants ranging 20–79 y/o (Acosta-Cabronero et al., 2016), between 20–90 y/o (Li Y. et al., 2021), 21–58 y/o (Burgetova et al., 2021), 25–78 y/o (Gong et al., 2015), 18–80 y/o (Lao et al., 2023), 50–80 y/o (Li J. et al., 2021), 10–70 y/o (Li et al., 2023), 20–69 y/o (Liu et al., 2016; Persson et al., 2015), 69–86 y/o (Poynton et al., 2014). When applying a linear regression model to our data, it can be observed that the slopes exhibit similar positive trends in terms of iron quantification values with respect to age. This can also be evidenced visually, from $R_{2}^{*}$ and QSM images, as the voxels' intensity is higher, particularly for the putamen and caudate regions (see Figure 1).

Regarding the thalamus, a negative small slope has been described in the literature (Burgetova et al., 2021; Gong et al., 2015; Li Y. et al., 2021; Li J. et al., 2021), with which our results are also consistent. Notice that more similar values of slope are obtained when the age range of the cohorts are alike. In fact, the iron load changes at different rates through lifespan (Treit et al., 2021), therefore linear curve fitting should be performed by specific age ranges. For instance, it has been described that the thalamus presents an increase of iron load until the fourth decade of age, which then decreases (Burgetova et al., 2021). Moreover, results presented in our work suggest that for some regions (amygdala, thalamus and globus pallidus) there are differences between male and female participants, that should also be taken into account when computing models. From all the studied ROIs, all but the hippocampus exhibit differences, which are either age and/or sex related. This is why normality values should be compared considering these two variables.

In our analysis we also considered risk factors that are common in old age (such as diabetes, high blood pressure and dyslipidemia). Although our cohort is particularly healthy and only a few present certain comorbidities at some extent, we noticed that even if these are treated, differences can be observed in QSM and $R_{2}^{*}$ values (*e.g*. for the putamen and globus pallidus when having at least 2 as risk factors score compared to 0, according to our score). Higher susceptibility values have been previously reported in the putamen and caudate in association with diabetes type 2, as well as in the thalamus for smokers (Li J. et al., 2021). Nevertheless, the relationship between cardiovascular risks and iron accumulation is not yet fully understood. Some hypotheses point to an iron accumulation due to microvascular hemorrhages (Li et al., 2020). Moreover, some suggest that brain iron is highly modulated by the diet (Hagemeier et al., 2015). BMI has also been previously reported to be related with higher iron levels measured by means of $R_{2}^{*}$ in the caudate, putamen and globus pallidus (Holz et al., 2022) and the amygdala and hippocampus (Pirpamer et al., 2016). From our study we evidenced this difference particularly in the putamen of female participants by means of both QSM and $R_{2}^{*}$.

In general, the obtained values for QSM and $R_{2}^{*}$ present a high variability (as it can be observed in the standard errors from the linear regressions). These variations can stem from a variety of sources, such as the inherent variability in the physiology of each individual subject, or coming from the acquisition parameters. In fact, the choice of imaging parameters can impact the quality of the measure, for instance partial volume effects due to a low resolution can have a great impact specially when measuring small structures as the DGM. Nevertheless, from our results there is a high correspondence between values obtained from QSM and $R_{2}^{*}$, which has been already described in the literature (Feng et al., 2018; Peterson et al., 2019; Deistung et al., 2013; Ghassaban et al., 2019). This confirms that the reconstructed QSM maps through our robust pipeline, although small singularities can still be noted specially near the sinuses, behaves as expected in regard of $R_{2}^{*}$, particularly for the DGM measurements. The reliability of the QSM reconstructions was also confirmed regarding contralateral structures, specially for those located in areas more prone to singularities (Supplementary Section 3.2). A great advantage of our pipeline is that it can be applied to any other database with MGRE acquisitions at its disposal.

As it is described by Wang et al. (2020), $R_{2}^{*}$ relates to compartmentalized “inclusions” with a susceptibility offset compared to the surrounding tissue, which broaden the distribution of frequencies within a voxel. The GRE signal phase is driven by an average magnetic susceptibility in a voxel, which relates to the relative size of each susceptibility-shifted compartment. Thus, on one hand, the QSM and $R_{2}^{*}$ contrasts rely essentially on the phenomenon, on the other hand they use different information and reconstructions to be extracted. From this statement, it is not surprising to see QSM and $R_{2}^{*}$ techniques exhibit a similar behavior for most of the regions and a high correlation between these two proxies have been previously documented (Feng et al., 2018; Ghassaban et al., 2019).

Our results are in line with this concordance between QSM and $R_{2}^{*}$. In addition, we evidenced that they could also provide complementary information, as differences could be reflected by one or the other proxy. In fact, our results showed differences between these two techniques regarding the sex-based analysis. While we were able to detect sex-related differences for some regions in QSM and $R_{2}^{*}$, these regions differ between them. For QSM, differences were found for the thalamus and amygdala regions, as for $R_{2}^{*}$ these were found for the globus pallidus. Moreover, we evidenced the joint effect of age and sex in regions such as the accumbens and hippocampus, suggesting that these two factors should be taken into account when performing a detailed analysis of the iron profile by means of both QSM and $R_{2}^{*}$. Sex-based differences in the brain iron load are still a source of debate. Sex-related differences have been already described in the literature for young subjects, from $R_{2}^{*}$, signaling lower iron deposition in boys than girls for several brain regions (Peterson et al., 2019). A lower ferritin dependence has been reported in women in regions such as the caudate and the thalamus (Bartzokis et al., 2007). Evidence of this sex-based differences has been highlighted by means of QSM for the thalamus (Gong et al., 2015), putamen, red nucleus and substantia nigra (Persson et al., 2015; Li et al., 2023), for which female subjects present lower susceptibility values. On the other hand, some studies have also reported no influence of sex in susceptibility values (Gong et al., 2015; Li J. et al., 2021; Holz et al., 2022; Li et al., 2015).

The underlying reasons of sex-based iron differences are not yet clear. Our data showed that hematocrit is higher in male than in females. As we noted both higher or lower QSM values in females, our data can not elucidate any relationship between systemic iron and brain iron measured by QSM. Previous studies often point to hormonal differences due to estrogen and to menstruation, as differences are related with menarche and menopause (Grubić Kezele and Ćurko-Cofek, 2020; Larsen et al., 2023).

Regarding our genetic analysis, we only included the presence of the ApoE ϵ4 allele, as it is the only one for which there is consensus as a risk factor in developing Alzheimer's disease. Therefore, it is interesting evaluating its influence in the predisposition in iron accumulation in healthy subjects. We evidenced that the presence of ϵ4 allele does not modify the iron accumulation in the DGM, which has also been previously reported (Li J. et al., 2021). Nevertheless, higher susceptibility has also been reported in the hippocampus and amygdala for carriers of ApoE ϵ4 allele, however this is also the case for ApoE ϵ2 carriers, particularly those under 65 y/o (Nir et al., 2022).

Regardless of the high quality of the data and the robustness of the pipeline, some limitations were present during our study. First, due to an MRI protocol update the sample size was reduced to a fraction of the total cohort, which limits the general population representation in order to establish a norm. This also limited our study to a cross-sectional analysis and prevented us from performing a longitudinal one at the current date. New time points are currently being acquired, therefore future research using our cohort will be centered in investing brain iron accumulation using a longitudinal design. Additionally, in our cohort, only some participants present an ApoE ϵ4 allele, which affects the statistical power of our analysis in this matter. Moreover, only one participant was homozygous for this allele. Small subgroup size presents also a limitation when performing statistical analysis for the participants with a high CRS, as our cohort is particularly healthy with only a few presenting comorbidities linked with an increase of cardiovascular risk. Another limitation regarding the CRS is the available information to be included when calculating the score. For instance, it might also be relevant to include information regarding the physical state and diet (Sacco, 2011). Moreover, our cohort is a particular case of a very healthy elderly population, which does not necessarily reflect the general population of older adults, therefore the results should be interpreted accordingly. Although the participants are biologically well-characterized, some parameters of interest such as serum ferritin and transferrin were not available. Its inclusion in future analysis should be considered, as $R_{2}^{*}$ values in some DGM regions have been described to be correlated with serum transferrin in older subjects with no cognitive impairment (House et al., 2010). It is also important to note that quantitative results are difficult to directly compare between studies, due to the inherent differences in QSM and $R_{2}^{*}$ maps that can arise from multi-centric bias (due to the machine, acquisition parameters and/or pipelines used). In this case, only the trend of the values extracted from the maps, as well as the differences between groups given a parameter, should be compared.

Moreover, due to our multiple statistical test, in order to reduce the type I risk (false positive test) we performed a Bonferroni correction. This led to decreased statistical power, as many of the differences caught by the tests do not pass this correction. Furthermore, it is worth keeping in mind that this type of correction can also increase type II risk (false negative test). Notice also the drastic reduction of the difference's significance by the Bonferroni correction might be also due the rather subtle variation of the factors evaluated, especially those related to cardiovascular risks given the healthy status of our cohort and the control of comorbidity by medical treatments, such as for arterial hypertension or diabetes.

Furthermore, this work was focused only in the study of most of the DGM, as many of these structures have been reported of having a particular incidence in specific pathologies. Even if other regions, such as the substantia nigra, would have been of major interest to segment and to study, they were not included in this work due to limitations in the segmentation tool used, which did not include this feature. Other available solutions require higher spatial resolutions (Manjón et al., 2020) or multi-contrasts (Langley et al., 2015; Xiao et al., 2012) that were not acquired in SENIOR cohort. Also, the gray matter arranged in the cortex has also been described to play a role in this matter. Susceptibility differences between controls and patients have been reported in the cortex, either in specific regions (*e.g*. in Parkinson's disease) or in a diffuse way all over it (*e.g*. in multiple sclerosis) (Ravanfar et al., 2021; Cohen-Adad et al., 2011). Despite the fact that cortical susceptibility information is essential for the better understating of these pathologies, QSM cortical reconstructions are still a challenge, essentially due to the *B*_0_ field inhomogeneities (Cohen-Adad et al., 2011). Despite the limitations found in the input phase data (see Supplementary Figure S2), thanks to the developed QSM computation pipeline it was possible to reconstruct the values for the deep gray matter structures. However, cortex values are less reliable and often cropped out of the QSM maps, specially close to the brain boundaries. Additional phase pre-processing would be needed for mitigating the *B*_0_ inhomogeneities effects in these boundary regions.

The developed pipeline allowed the addition of the iron load, measured by two proxies (QSM and $R_{2}^{*}$), as a biomarker to the SENIOR database. This made possible the establishment of a range of normal values during healthy aging for our cohort and evaluate if common comorbidities have an impact in the measurements. This allowed the delineation of a general trend for each region, reflecting the iron level changes during aging. Regardless of the inherent batch variability across studies mentioned before, these general trends could be useful for comparison purposes for other studies. Moreover, as some previous studies have also done, we show that iron load values should be evaluated according to the age and sex of the participants, as these two variables play a major role in the differences that are found between them. In addition to this information, we also provide insights regarding other parameters that should be looked into (CRS and BMI). High quality data and the use of QSM and $R_{2}^{*}$ allow the detection of differences in the iron load that are proven to be significant, even though these are subtle. Notice that even if several of our results are in line with the literature, no gold standard values for either $R_{2}^{*}$ nor QSM have been established. Therefore, results should be interpreted carefully, specially keeping in mind the variability of these two proxies. Following work will be focused in including in the study participants presenting Alzheimer's disease to assess the ability to detect differences with respect to our healthy participants. Moreover, as new time-points are being added to the database, a next step will be moving to a longitudinal study design.

## Data Availability

The data that support the findings of this study are available from the corresponding author, AV, upon request.

## Footnotes

^1^ https://volbrain.upv.es/
