# Supplementary material for: Iron load in the normal aging brain measured with QSM and R2* at 7T: findings of the SENIOR cohort
Source: Front Neuroimaging. 2024 Oct 21;3:1359630. doi: 10.3389/fnimg.2024.1359630 (PMC11533018; doi:10.3389/fnimg.2024.1359630)
Supplement: Supplementary file 1 [file Data_Sheet_1.pdf]

Supplementary Material

1 PARTICIPANTS INCLUSION AND EXCLUSION CRITERIA

The inclusion and exclusion of the participants for the SENIOR study were carefully evaluated according to the criteria mentioned below.

1.1 Exclusion or non-inclusion criteria

- Neurological or psychiatric history.
- Recent or severe cardiovascular history (less than one year).
- Poorly controlled arterial hypertension.
- History of serious, severe or unstable pathologies.
- History of alcoholism or illicit substance use in the previous two years.
- Abnormalities detected on inclusion MRI: stroke, tumor, head trauma, hydrocephalus, etc.
- Artifacts detected on inclusion MRI (*e.g.* related to dental amalgams or crowns) inside the Volume Of Interest (VOI) and interfering with image analysis.
- Subject concerned by the restriction of categories (book of the public health code for research without direct individual benefit).
- Failure to sign an informed consent form.
- Non-affiliation with a Social Security scheme.
- In a period of exclusion.
- Major physical problems likely to interfere with tests (sight, hearing, etc.).
- Pregnancy.

1.2 Additional exclusion criteria for MRI

- Implants (mechanical or electronic: cochlear implants, pacemakers, infusion pumps, magnetic aneurysm clips, etc.) incompatible with the magnetic field.
- Metallic foreign bodies in the eye or nervous system.
- Non-removable metal objects likely to concentrate the radiofrequency field (*e.g.* piercings).
- Claustrophobia or severe anxiety.
- Unlikely cooperation.
- Non-removable dental devices.
- Tinnitus.
- Colored tattoos (depending on the pigments used).
- Overweight patients who are unable to settle comfortably in MRI scanners.

1.3 Inclusion criteria

To be included, the subject must :

- Present no abnormalities on the pre-inclusion 3T MRI scan.

- Be at least 50 and less than 70 years of age.
- Be affiliated with or benefit from a social security scheme.
- Sign an informed consent and agree to participate in this study for 10 years.
- Have no spontaneous memory complaint.
- Obtain normal scores on global intellectual efficiency tests (MMS greater than 24).
- Have French language proficiency for test taking.

2 POPULATION CHARACTERISTICS.

Detailed description of the seventy-seven participants used in the study for the population analysis, selected from the SENIOR cohort. A general global information about the participants is given in Table S1

Table S1. Population general characteristics summary.

	Sex	54-62 y/o	62-69 y/o	69-79 y/o
Total number of participants	♀	9	14	17
	♂	15	12	10
Age median (min-max)	♀	59.31 (57.99 - 61.9)	65.43 (62.36 - 68.03)	73.3 (69.41 - 77.72)
	♂	58.18 (54.67 - 61.95)	64.31 (62.01 - 68.35)	72.8 (70.93 - 78.95)
BMI ($^{Kg}/_{m^2}$) median (min-max)	♀	21.30 (18.25 - 24.97)	21.83 (17.31 - 32.41)	25.32 (18.90 - 29.24)
	♂	24.45 (21.07 - 38.28)	24.12 (22.28 - 34.23)	24.05 (21.41 - 32.49)
Total number smokers	♀	1	1	1
	♂	0	0	1
MMS median (min-max)	♀	30 (29 - 30)	30 (28 - 30)	30 (26 - 30)
	♂	30 (27 - 30)	29.5 (28 - 30)	30 (28 - 30)
GDS median (min-max)	♀	3 (0 - 14)	2.5 (0 - 12)	4 (0 - 12)
	♂	4 (0 - 17)	1.5 (0 - 6)	3.5 (0 - 7)
Number of depressed participants	♀	1	2	2
	♂	1	0	0
Mattis median (min-max)	♀	144 (142 - 144)	144 (143 - 144)	144 (142 - 144)
	♂	144 (143 - 144)	144 (143 - 144)	144 (139 - 144)

Demography summary and neuropsychologic and neuropsychological testing results. It presents the distribution of the population regarding the age and sex and their cognitive state. For each parameter the median, minimum and maximum values are given. Threshold for a participant to be considered as depressed: GDS$\geq$10. BMI: Body Mass Index, MMS: Mini-Mental State examination, GDS: Geriatric Depression Scale and Mattis: Mattis Dementia Rating Scale.

The presence of depression in the participants was evaluated using the Geriatric Depression Scale (GDS) (Yesavage et al., 1982), defined in French by the GRECO (Groupe de Reflexion sur les Evaluations Cognitives). All participants present a GDS$<$20, therefore none of them were considered to have a severe depression, consequently none of them were excluded from the study.

Table S2. Population lipid balance summary.

	Sex	54-62 y/o	62-69 y/o	69-79 y/o
Number of participants with cholesterol disorder	♀<br>♂	2<br>2	7<br>1	6<br>1
HDL (g/L) median (min-max)	♀<br>♂	0.68 (0.63 - 0.92)<br>0.51 (0.34 - 0.78)	0.77 (0.4 - 1)<br>0.55 (0.4 - 0.85)	0.83 (0.5 - 1.18)<br>0.61 (0.48 - 0.87)
LDL (g/L) median (min-max)	♀<br>♂	1.33 (1.22 - 1.7)<br>1.52 (0.66 - 1.87)	1.46 (0.92 - 2.08)<br>1.22 (0.76 - 1.62)	1.24 (0.92 - 2.46)<br>1.25 (0.49 - 1.53)
Triglyceride median (min-max)	♀<br>♂	0.72 (0.42 - 1.25)<br>1.29 (0.61 - 2.02)	0.88 (0.6 - 1.61)<br>0.88 (0.53 - 1.44)	0.92 (0.48 - 1.67)<br>0.98 (0.61 - 1.8)
$HDL + LDL/HDL$ median (min-max)	♀<br>♂	1.5 (1.37 - 1.75)<br>1.36 (1.22 - 1.62)	1.5 (1.19 - 1.88)<br>1.39 (1.32 - 1.8)	1.68 (1.26 - 1.91)<br>1.5 (1.41 - 2.21)
$Triglyceride/HDL$ median (min-max)	♀<br>♂	1.11 (0.46 - 1.87)<br>2.35 (0.9 - 5.05)	1.24 (0.7 - 4.03)<br>1.55 (0.62 - 2.82)	1.1 (0.49 - 2.94)<br>1.67 (0.7 - 3.16)

Lipid balance of the participants. It presents the values from the cholesterol measures from blood samples (median, minimum and maximum values). A participant is considered to present cholesterol disorder if: blood cholesterol > 2.5 ($mmol/L$) or is under treatment or self-reported the disease.HDL: High-Density Lipoprotein, LDL: Low-Density Lipoprotein.

Table S3. Population systemic blood iron balance summary.

	Sex	54-62 y/o	62-69 y/o	69-79 y/o
Red blood cells ($10^6/mm^3$) median (min-max)	♀<br>♂	4.53 (4.24 - 4.89)<br>4.9 (4.43 - 5.27)	4.44 (3.92 - 4.89)<br>4.86 (4.19 - 5.57)	4.36 (4.02 - 4.82)<br>4.72 (4.4 - 5.44)
Hemoglobin (g/dL) median (min-max)	♀<br>♂	13.4 (13.1 - 14)<br>14.7 (13.8 - 16)	13.45 (12.5 - 15)<br>14.85 (11.4 - 16.2)	13.7 (12.6 - 14.8)<br>14.95 (13.7 - 16.1)
Hematocrit (%) median (min-max)	♀<br>♂	39.8 (38.7 - 42.3)<br>43.8 (39.6 - 46.5)	40.1 (36 - 45.7)<br>43.45 (36.9 - 46.4)	40.3 (37.9 - 43.2)<br>45.7 (40.2 - 46.8)
MCV (fL) median (min-max)	♀<br>♂	87.6 (85.9 - 94.7)<br>88.2 (85.5 - 94.7)	89.75 (86.8 - 98.8)<br>90.45 (66.2 - 96.4)	93.3 (86.6 - 98.6)<br>91.75 (85.9 - 100.4)
MCHC (g/dL) median (min-max)	♀<br>♂	33.7 (32.9 - 34)<br>34.3 (33.1 - 34.8)	33.65 (32.8 - 34.6)<br>34.2 (31 - 35)	33.5 (32.2 - 34.3)<br>34.95 (33.4 - 34.7)
MCH ($fmol/cell$) median (min-max)	♀<br>♂	29.2 (28.8 - 31.2)<br>30.1 (28.3 - 32)	30.2 (29 - 33.8)<br>31 (20.5 - 33.2)	31.3 (29.4 - 33.3)<br>30.9 (29.6 - 34.6)

Systemic blood iron balance of the participants. It presents the values from hemoglobin measures from blood samples (median, minimum and maximum values). MCV: Mean Corpuscular Volume, MCH: Mean Corpuscular Hemoglobin, Mean Corpuscular Hemoglobin Concentration.

3 QSM AND R_2^* COMPUTATION PIPELINE.

A pipeline was implemented in Python, nobly using using Common Work Flow Language to compute R_2^* and QSM maps, and extract values for several regions of interest (ROIs) automatically. A schematic of the pipeline is presented in Figure S1. The pipeline seeks to overcome possible artifacts in the input data phase data (*i.e* phase combination method, presence of phase singularities [often referred to as open-ended fringe lines], etc). See Figure S2 for an example of the QSM maps computed with and without the pipeline.

Table S4. Population blood glucose balance summary.

	Sex	54-62 y/o	62-69 y/o	69-79 y/o
Number of participants with diabetes	♀	0	0	1
	♂	0	0	0
Blood glucose ($^g/_{dL}$) median (min-max)	♀	0.88 (0.86 - 1)	0.91 (0.76 - 1.05)	0.97 (0.77 - 1.29)
	♂	0.99 (0.74 - 1.08)	0.99 (0.87 - 1.24)	1.04 (0.84 - 1.15)
Hb1AC (%) median (min-max)	♀	5.4 (4.8 - 5.7)	5.5 (5.1 - 5.8)	5.4 (5 - 5.9)
	♂	5.6 (5.2 - 5.9)	5.45 (5.2 - 6.1)	5.5 (5.2 - 6)

Glucose balance of the participants. It presents the values from the glucose measures from blood samples (median, minimum and maximum values) and HbA1c: Glycated hemoglobin.

Table S5. Population hypertension summary.

	Sex	54-62 y/o	62-69 y/o	69-79 y/o
Number of participants with hypertension	♀	1	4	5
	♂	2	2	5
SBP	♀	116.0 (102.0 - 147.0)	128.0 (111.0 - 162.0)	131.0 (112.0 - 155.0)
	♂	134.0 (110.0 - 191.0)	137.5 (96.0 - 168.0)	151.5 (108.0 - 192.0)
DBP	♀	75.0 (67.0 - 93.0)	73.5 (56.2 - 92.0)	72.0 (55.0 - 85.0)
	♂	86.0 (66.0 - 100.5)	72.5 (50.0 - 108.0)	85.0 (67.0 - 914.0)

Hypertension status of the participants, from blood pressure measurements. It presents the number of participants that present hypertension (Systolic: SBP and Diastolic: DBP).

Table S6. Population ApoE summary.

ApoE	$\epsilon3/\epsilon4$		$\epsilon3/\epsilon3$		$\epsilon2/\epsilon3$		$\epsilon2/\epsilon4$		$\epsilon4/\epsilon4$	
Age \ Sex	♀	♂	♀	♂	♀	♂	♀	♂	♀	♂
54-62	2	4	6	10	1	1	0	0	0	0
62-69	3	2	11	7	0	2	0	1	0	0
69-78	3	0	10	8	4	1	0	0	0	1

Population ApoE distribution according to age range and sex. No participants with the $\epsilon2/\epsilon2$ configuration were present in the cohort.

The pipeline essentially takes as inputs multi-gradient-echo acquisition (MGRE) and MP2RAGE DICOM data, as well as an externally provided segmentation in Nifti format. The segmentation was done using volBrain[1] (Manjón and Coupé, 2016) based on the MP2RAGE Uniform Denoised (UNIDEN) volume. All segmented volumes were reviewed beforehand by a trained expert radiologist among the authors.

A first check for completion of DICOM files was performed before converting them to Nifti format using dcm2niix (Li et al., 2016), and concatenating the multiple MGRE data to a single NumPy array (Harris et al., 2020) data format.

For the R_2^* map computation, a Gauss-Netwton algorithm was implemented, in order to perform a non-linear fit for the magnitude from the ten available echoes. The implementation fits voxel-wize the

[1] https://volbrain.upv.es/

signal evolution as a function of echo index k as $A \times B^k$, where A is the fitted magnitude for $k = 0$ and $B = exp(-R_2^* \times \delta TE)$ with δTE the constant echo spacing.

For the QSM computation, phase maps must be exploitable even when the input phase data present singularities, which were observed in our dataset. In order to achieve this, the method integrates a pre-filtering of the phase data from the MGRE acquisition by using the information of the magnitude and phase from the ten echoes. It is based on the method described in de Rochefort et al. (2009), where in order to isolate the brain internal field variations (ΔB_{in}) the conjugate gradients algorithm of the normal equation $\Delta W_{\Delta}^2 \Delta B_{in} = \Delta W_{\Delta}^2 \Delta B$ is computed, where W_{Δ} is a diagonal matrix weighting each estimation of the Laplacian by the inverse of its error standard deviation. The unwrapping is included in the analysis by forcing the point by point difference between]-π, π] when calculating ΔB using the modulo function. The processing starts with the computation of the Laplacian of the field. To this aim, the Laplacian of the phase for each echo k is computed (ΔB_k), as well as its error ($W_{\Delta k}$), before being combined in the least-squares sense. The gradient norm of the field was also computed similarly.

A mask containing only the voxels with reliable information was then computed. It stems from a whole-brain mask refined using the information from the gradient of the field. The whole-brain mask is computed using Advanced Normalization Tools (ANTs) from the T_1-weighted MP2RAGE UNIDEN volume. The MP2RAGE is registered using a rigid transformation to the magnitude of the MGRE first echo, and this transformation is then applied to the mask. The gradient norm of the field is used to remove voxels suffering from spatial deformation (such as the region near the sinus) which lead to a ΔB_{in} that cannot be correctly measured. A first restriction of the analysis to the area inside the brain is performed for the gradient norm, given by the whole-brain mask. Then, a thresholding step keeps only the reliable voxels. Morphological operations aiming to eliminate isolated voxels and the filling of holes (opening, connected components algorithm and closing) are then applied. At last, the conjugate gradient algorithm is used to compute B_{in} from the normal equation (de Rochefort et al., 2009). Iterations were stopped when maximum number of iterations is reached (512) or the relative norm was less than 10^{-3}.

For the QSM computation per-se, we used QSMetric - research edition (MedImageMetric LLC, NY, USA, provided by Ventio, Marseille, France), an automated implementation of MEDI (de Rochefort et al., 2010; Liu et al., 2011b, 2012; Spincemaille et al., 2019). The software computes the QSM maps from the filtered phase data, using as reference value the cerebrospinal fluid (CSF) in the ventricles.

To determine R_2^* and QSM ROI values, the segmented labelled volumes were propagated to the MGRE space.

The pipeline was deployed in several ISO27001-certified cloud computing environments implementing openstack, in particular on the German Network for Bioinformatics Infrastructure (de.NBI cloud). The computing resources automatically deployed corresponded to virtual machines running under Ubuntu Linux version 20.04 with 14 cores, 32 Go RAM and 200 Go volumes.

3.1 QSM and R_2^* maps reproducibility.

We tested the reproducibility of the values obtained from the pipeline described in Section 3 if it was run more than once over different acquisitions of the same subject. In order to do that, a participant was scanned three times in a row with the MGRE acquisitions without repositioning, generating three different input datasets. Then, the pipeline was applied to each independent input dataset, generating three QSM maps and three R_2^* maps. We computed the voxel-by-voxel absolute differences between each pair, followed by the average absolute differences between the three pairs. We then evaluated the mean and standard deviation of

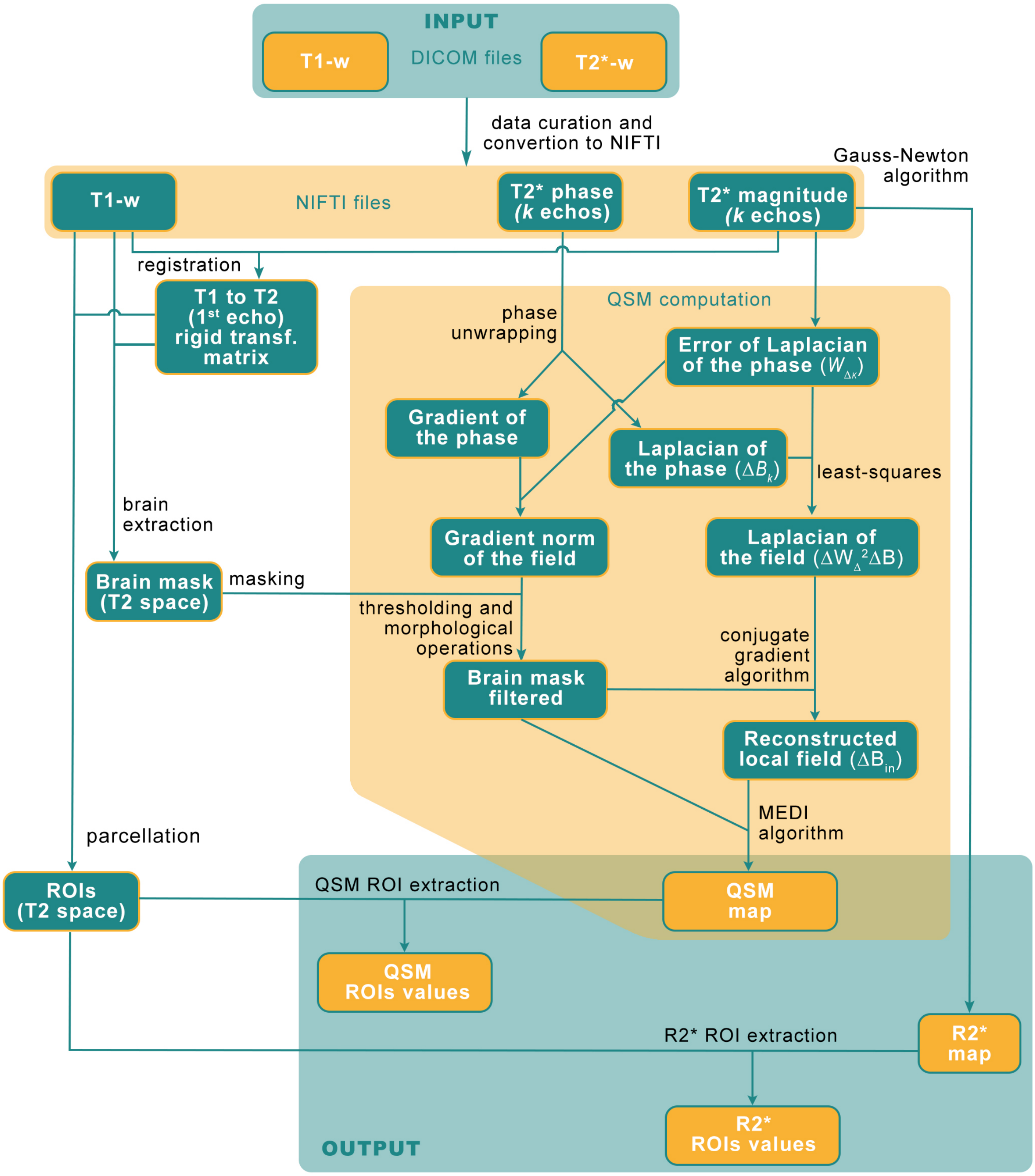

Figure S1. Diagram of the implemented pipeline for the computation of QSM and R_2^* maps. For the computation of QSM, it includes the pre-processing steps for the reduction of phase singularities.

the absolute difference in the whole brain, as well as for each isolated ROI in order to see if any of them is more prone to yield different results (see Table S7). From the results it can be seen that for all the DGM ROIs and all the maps the mean absolute difference is close to zero. Inside the ROIs, the standard deviation of the difference was always lower than 1 ppb (for QSM) and $0.4s^{-1}$ (for R_2^*). As could be expected for the whole brain, differences were higher particularly in the areas near the borders (cortex), which is reflected by a higher mean and standard deviation of the absolute differences.

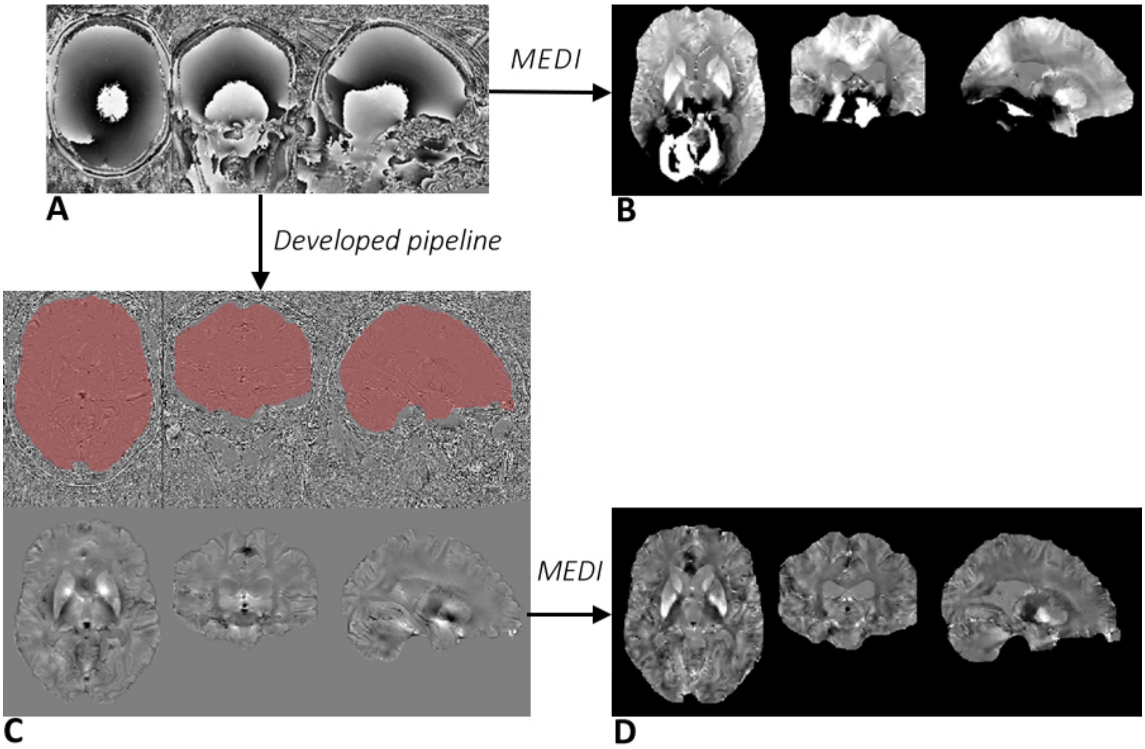

Figure S2. QSM reconstruction with and without our developed pipeline. The input phase image (A) presents singularities as open-ended fringe lines which are translated into errors in the QSM reconstruction (B) if MEDI is applied without any pre-processing (using projection-onto-dipole field, (Liu et al., 2011a)). Thanks to our pipeline, by the computation of the Laplacian of the phase and a filtering mask (C first row), a resulting filtered field map (C second row) is obtained (from the conjugate gradients calculation). This allow obtaining a proper QSM reconstruction (D) from the internal field.

3.2 QSM maps reliability test

In order to verify the reliability of the QSM maps, specially in regions prone to present singularities, for all the studied DGM regions we performed a correlation analysis. For each DGM we evaluated the correlation between the left and right hemispheres, as well as the correlation between the left or right hemisphere versus the joint values of both hemispheres (*i.e.* left and right hemisphere ROIs are considered as a single ROI). The results of these correlation tests are presented in Table S8. The mean and standard deviation values are also included to inform of the variability present in each ROI. R_2^* values are also presented for reference. It can be seen that for all the regions, when comparing each hemisphere separately and the joint region there are no significant changes regarding the mean and standard deviation values, even for DGM structures more susceptible to artifacts such as the amygdala and the hippocampus. Notice that the absence of significant differences between hemispheres (from the Mann-Whitney U test) lead us to consider both hemispheres as a single ROI for posterior statistical analysis. Regarding the correlation between hemispheres, when comparing just left and right, it can be seen that regions more susceptible to artifacts are indeed poorly correlated. However, when comparing each hemisphere with the joint region, all regions present a considerably higher correlation. In fact, regions that are more prone to artifacts in QSM can be modified in terms of ROI volume by our pipeline, in order to exclude voxels where the QSM values are invalid. This could explain why when comparing only the left and right hemisphere, we obtain a lower correlation. This is not the case for R_2^* values which are more stable and therefore the ROI volumes are kept without changes, and therefore exhibiting a higher correlation. Moreover, when joining the two

Table S7. Differences from the reproducibility test.

ROI	Hemi	QSM (ppb) $\overline{X}$	QSM (ppb) S	R_2^* (s^{-1}) $\overline{X}$	R_2^* (s^{-1}) S
Caudate	L	0.01	0.38	0	0.19
	R	0.01	0.44	0	0.3
Putamen	L	0.01	0.4	0	0.21
	R	0.01	0.44	0	0.22
Thalamus	L	0.01	0.3	0	0.21
	R	0.01	0.3	0	0.21
Globus Pallidus	L	0	0.22	0	0.17
	R	0	0.36	0	0.15
Hippocampus	L	0.01	0.57	0	0.32
	R	0.01	0.49	0.01	0.36
Amygdala	L	0	0.19	0	0.08
	R	0	0.16	0	0.1
Accumbens	L	0	0.08	0	0.03
	R	0	0.12	0	0.03
Whole brain	-	2.6	9.92	1.58	8.71

Absolute differences for QSM and R_2^* maps obtained from three consecutive acquisitions of a single subject. For each proxy, the three datasets were compared between them for all the ROIs and the whole brain and then averaged to a single value ($\overline{X}$). The standard deviation (S) was also computed.

hemispheres into one ROI, it stabilizes the values by adding more information stemming for a greater number of voxels. The reliability of the maps can then be evaluated by the comparison of each hemisphere to the common joint ROI, which is satisfactory.

4 CARDIOVASCULAR RISK SCORE.

The cardiovascular risk was scored on a scale from 1 to 5 the risk of developing a cardiovascular disease based on factors that have an impact on it.

5 DIFFERENCES FOR THE TESTED VARIABLES.

A summary of the results obtained for the values of QSM and R_2^* for the age-, sex-, CRS- and ApoE $\epsilon4$ allele-based groups is presented in Table S10.

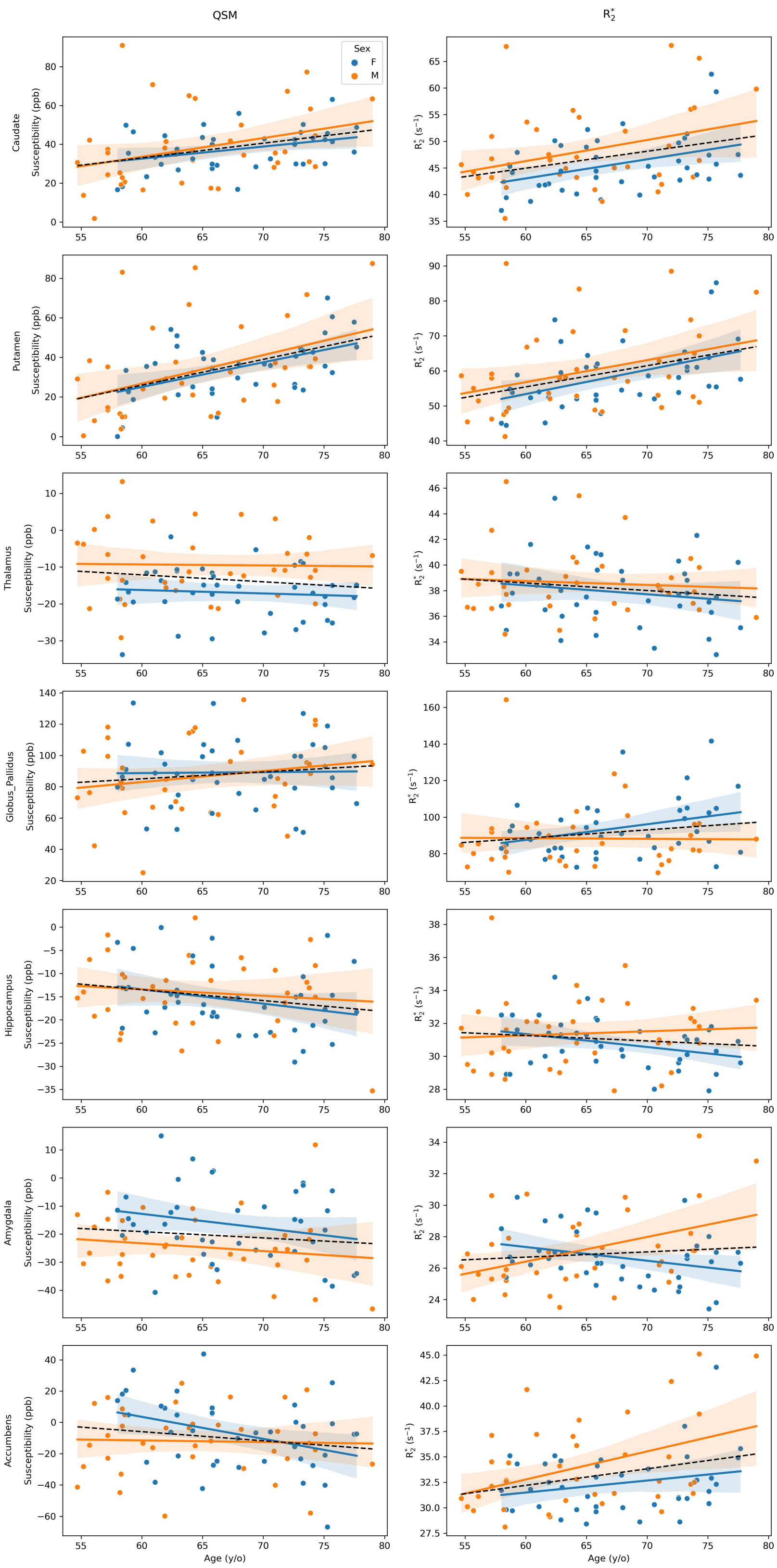

Figure S3. Scatter plots and linear regressions for all the analyzed regions for both QSM and R_2^*. For each region the linear regressions are presented for male (orange) and female (blue) participants separately, with a confidence interval of 95%. A black line representing the linear regression for the whole population (male and female participants together) is also included

Table S8. Hemisphere values validation.

		QSM					R_2^*				
	hemisphere	mean	std	std/mean	L vs R	L or R vs joint	mean	std	std/mean	L vs R	L or R vs joint
	Left	36.27	12.08	0.33	0.80	0.95	46.54	4.97	0.11	0.98	0.99
Caudate	Right	39.19	12.53	0.32		0.95	47.28	5.16	0.11		0.99
	Joint	37.65	11.54	0.31			46.89	5.05	0.11		
	Left	34.05	15.52	0.46	0.93	0.98	59.38	8.41	0.14	0.98	0.99
Putamen	Right	33.94	14.04	0.41		0.98	58.92	7.83	0.13		0.99
	Joint	33.95	14.19	0.42			59.13	8.04	0.14		
	Left	-13.37	7.26	0.54	0.73	0.93	38.35	2.00	0.05	0.97	0.99
Thalamus	Right	-13.28	7.72	0.58		0.94	38.13	1.95	0.05		0.99
	Joint	-13.27	6.74	0.51			38.24	1.96	0.05		
	Left	86.09	20.82	0.24	0.75	0.94	91.25	12.12	0.13	0.98	0.99
Globus pallidus	Right	89.37	16.81	0.19		0.93	91.35	12.12	0.13		0.99
	Joint	87.72	17.43	0.20			91.26	12.02	0.13		
	Left	-14.58	7.65	0.53	0.43	0.85	31.15	1.45	0.05	0.82	0.95
Hippocampus	Right	-15.31	6.30	0.41		0.83	30.96	1.49	0.05		0.96
	Joint	-14.92	5.76	0.39			31.06	1.42	0.05		
	Left	-20.33	16.24	0.80	0.04	0.77	26.87	1.66	0.06	0.76	0.92
Amygdala	Right	-20.39	12.04	0.59		0.66	26.99	1.87	0.07		0.95
	Joint	-20.51	10.29	0.50			26.90	1.60	0.06		
	Left	-11.62	19.76	1.70	0.57	0.89	32.67	2.67	0.08	0.69	0.89
Accumbens	Right	-8.52	20.38	2.39		0.87	33.79	3.80	0.11		0.94
	Joint	-9.50	17.59	1.85			33.19	2.89	0.09		

For each DGM structure, the values for the hemispheres correlation analysis are presented. The values for each ROI and each hemisphere are given (for each DGM structure left and right hemispheres are considered independent ROIs), as well as their joint values (for one DGM structure the left and right hemispheres are considered as a single ROI). The mean and standard deviation values are informed under *mean* and *std* columns, respectively. The ratio between the standard deviation and the mean is also informed (*std/mean* column). Then, the correlation between the left and right hemispheres is informed under the *L vs R* column. Finally, the correlation of the left or right hemisphere ROI versus the joint hemispheres ROI is presented under the *L or R vs joint* column.

Table S9. Cardiovascular risk score.

Diabetes	Glycemia > 1.26 ($^g/_L$)<br>Treatment<br>Self-reported
High Blood Pressure	SBP > 140 $mmHg$ and DBP > 90 $mmHg$<br>Treatment<br>Self-reported
Dyslipidemia	Cholesterol > 2.5 ($^{mmol}/_L$)<br>Treatment<br>Self-reported
Smoking	Currently
BMI	> 30

Factors considered to calculate a cardiovascular risk score. If any of the conditions is present for each factor a value of 1 is assigned. Then the sum of all the values represent the cardiovascular risk score (0: low risk and 5: high risk). For the diabetes, high blood pressure and dyslipidemia, the normal range values are considered, or if the participant is under treatment or has informed the presence of the factor. SBP: Systolic Blood Pressure, DBP Diastolic Blood Pressure

Table S10. Summary of the results.

	Sex		Age		CRS		BMI		ϵx,4	
	QSM	R_2^*	QSM	R_2^*	QSM	R_2^*	QSM	R_2^*	QSM	R_2^*
Caudate	→	→	↗	↗	→	→	→	→	→	→
Putamen	→	→	↗*	↗*	↗	↗	↗♀	↗♀*	→	→
Globus Pallidus	→	↘	→	→	↗	↗	→	→	→	→
Thalamus	↗*	→	→	→	→	→	→	→	→	→
Hippocampus	→	→	→	↘♀	→	→	→	→	→	→
Amygdala	↘	→	→	→	→	→	→	→	→	→
Accumbens	→	→	↘♀	→	→	→	→	→	→	→

Summary of differences found in groups regarding the different metrics: age (older with respect to younger), sex (males with respect to females), age and sex (for females), CRS (high risk with respect to low risk), BMI (overweight with respect to to normal weight) and presence of one allele ApoE ϵ4 (with respect to its absence) . →: no difference, ↗: differences showing higher values in the first group versus the second one ($p \leq 0.05$ before Bonferroni correction); and ↘: differences showing lower values in first group versus the second one ($p \leq 0.05$ before Bonferroni correction). ♀: differences only for the female group. *: Regions showing statistically significant differences ($p \leq 0.05$) after Bonferroni correction.

REFERENCES

de Rochefort, L., Delzor, A., Guillermier, M., Houitte, D., Chaigneau, M., Déglon, N., et al. (2009). Quantitative susceptibility mapping in vivo in the rat brain. *Proc Intl Soc Magnet Reson Med* 17, 1134

de Rochefort, L., Liu, T., Kressler, B., Liu, J., Spincemaille, P., Lebon, V., et al. (2010). Quantitative susceptibility map reconstruction from mr phase data using bayesian regularization: validation and application to brain imaging. *Magnetic Resonance in Medicine: An Official Journal of the International Society for Magnetic Resonance in Medicine* 63, 194–206

Harris, C. R., Millman, K. J., van der Walt, S. J., Gommers, R., Virtanen, P., Cournapeau, D., et al. (2020). Array programming with NumPy. *Nature* 585, 357–362. doi:10.1038/s41586-020-2649-2

Li, X., Morgan, P. S., Ashburner, J., Smith, J., and Rorden, C. (2016). The first step for neuroimaging data analysis: Dicom to nifti conversion. *Journal of neuroscience methods* 264, 47–56

Liu, J., Liu, T., de Rochefort, L., Ledoux, J., Khalidov, I., Chen, W., et al. (2012). Morphology enabled dipole inversion for quantitative susceptibility mapping using structural consistency between the magnitude image and the susceptibility map. *Neuroimage* 59, 2560–2568

Liu, T., Khalidov, I., de Rochefort, L., Spincemaille, P., Liu, J., Tsiouris, A. J., et al. (2011a). A novel background field removal method for mri using projection onto dipole fields. *NMR in Biomedicine* 24, 1129–1136

Liu, T., Liu, J., de Rochefort, L., Spincemaille, P., Khalidov, I., Ledoux, J. R., et al. (2011b). Morphology enabled dipole inversion (medi) from a single-angle acquisition: comparison with cosmos in human brain imaging. *Magnetic resonance in medicine* 66, 777–783

Manjón, J. V. and Coupé, P. (2016). volbrain: an online mri brain volumetry system. *Frontiers in neuroinformatics* 10, 30

Spincemaille, P., Liu, Z., Zhang, S., Kovanlikaya, I., Ippoliti, M., Makowski, M., et al. (2019). Clinical integration of automated processing for brain quantitative susceptibility mapping: multi-site reproducibility and single-site robustness. *Journal of Neuroimaging* 29, 689–698

Yesavage, J. A., Brink, T. L., Rose, T. L., Lum, O., Huang, V., Adey, M., et al. (1982). Development and validation of a geriatric depression screening scale: a preliminary report. *Journal of psychiatric research* 17, 37–49
